# Supplementary figures and images for: An inhibitor-free, versatile, fast, and cheap precipitation-based DNA purification method
Source: PLoS One. 2025 Apr 8;20(4):e0317658. doi: 10.1371/journal.pone.0317658 (PMC11978010; doi:10.1371/journal.pone.0317658)

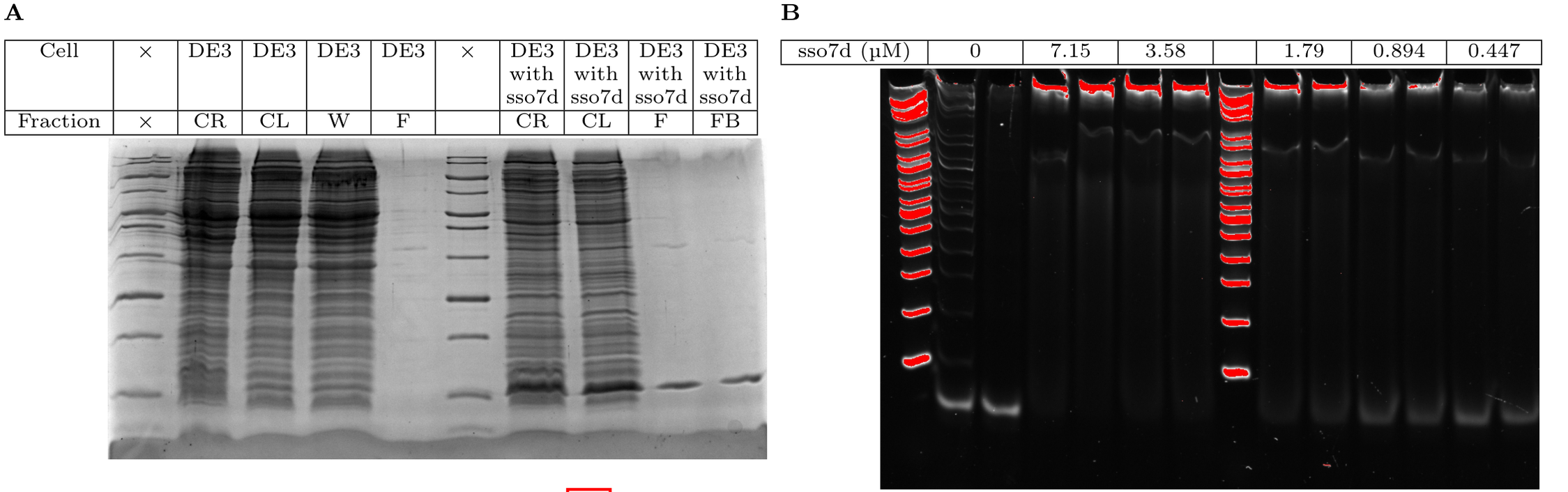

Supplement: S1 Fig — (A) SDS-PAGE analysis at various stages of the protein purification process. CR means crude lysate after sonication; CL means clarified lysate prior to loading to the Ni-TED column; W means the flowthrough fraction after the wash step; F means the final eluted protein sample; FB means the final protein sample after buffer exchange. (B) Electrophoretic mobility shift assay to detect sso7d binding of oligo duplex. (TIF) [file pone.0317658.s001.tif]

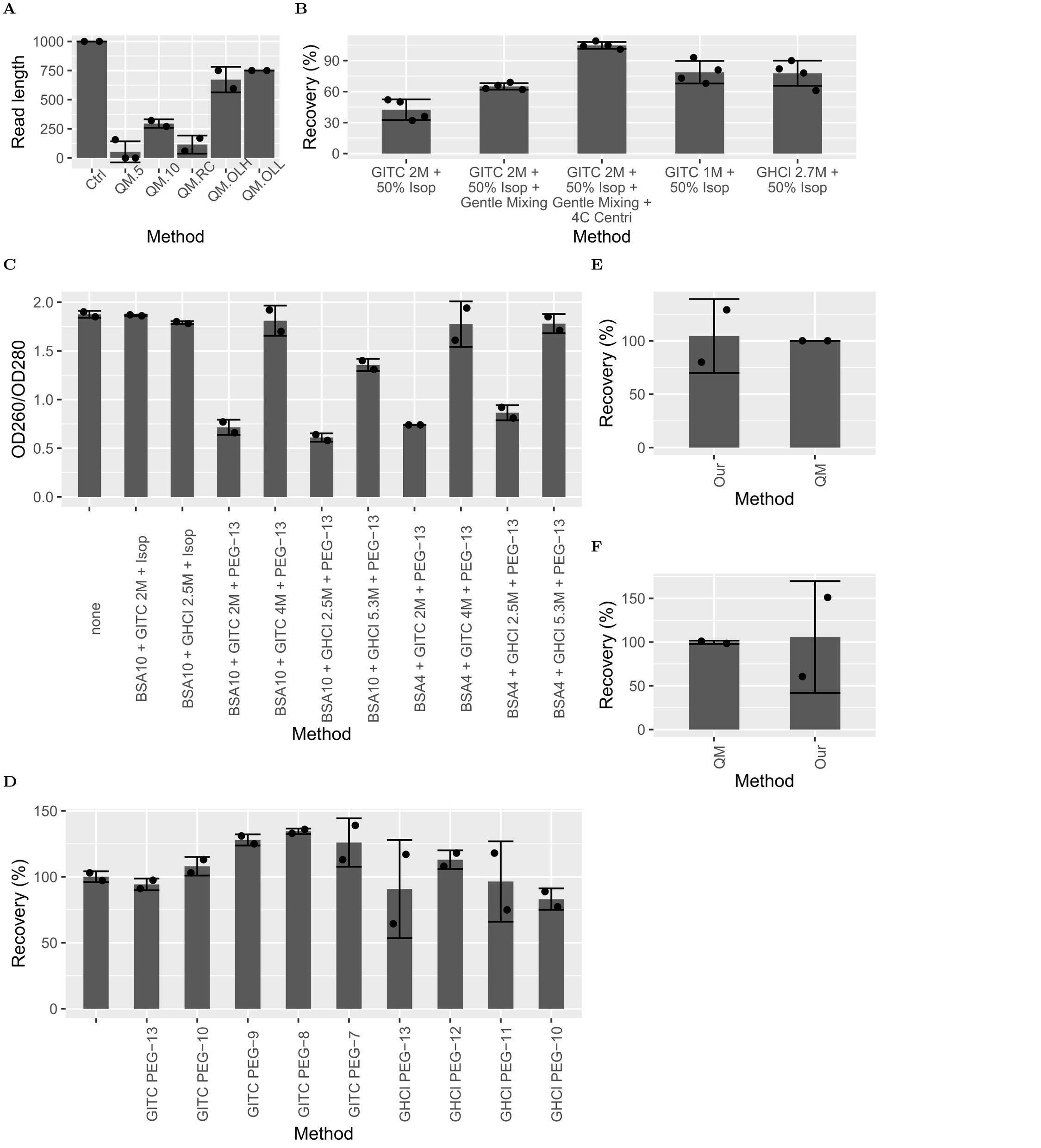

Supplement: S2 Fig — (A) Visualization of Fig 2A. (B) Visualization of Fig 2C. (C) Visualization of Fig 2F. (D) Visualization of Fig 3E. (E) Visualization of Fig 3F. (F) Visualization of Fig 3G. (TIF) [file pone.0317658.s002.tif]
