# Supplementary material for: An inhibitor-free, versatile, fast, and cheap precipitation-based DNA purification method
Source: PLoS One. 2025 Apr 8;20(4):e0317658. doi: 10.1371/journal.pone.0317658 (PMC11978010; doi:10.1371/journal.pone.0317658)
Supplement: Attachment — Submitted filename: Recommendation.pdf [file pone.0317658.s003.pdf]

The authors of paper entitled “An inhibitor-free, versatile, fast, and cheap precipitation-based DNA purification method” by Tang, Z. et al. propose a method using chaotropic salts and alcohol/polyethylene glycol for simultaneous protein deactivation and DNA precipitation. This method eliminates the need for multiple steps found in traditional techniques such as phenol-chloroform extraction and silica column absorption. However, here are a few potential areas of improvement as below:

1. Lack of Image Clarity:

Figures and Diagrams: Since you mentioned that the images are unclear, it’s important that the authors provide higher resolution or more detailed images. Figures such as the DNA electrophoresis results (gel images), and other experimental data are central to demonstrating the efficacy of the new method.

Recommendation: Authors should ensure that all figures are sharp and annotated clearly to highlight the key results. Increasing the image resolution or simplifying complex diagrams could improve reader comprehension.

2. Missing Quantitative Comparisons:

While the paper does mention that the new method is cheaper and faster, there are limited quantitative comparisons of yields, purity, or transformation efficiencies in the images, which could better illustrate the claimed advantages over other methods.

Recommendation: Provide side-by-side comparisons in the figures with additional data showing exact numbers (e.g., purity levels, transformation rates, yield efficiency), possibly through clearer charts or tables.

3. Incomplete Data Representation:

The article mentions certain experiments (like comparing the inhibition of T5 exonuclease) but doesn't fully visualize them clearly. The images used to represent these findings need to communicate the results effectively.

Recommendation: Consider adding more graphical elements like bar charts or scatter plots to visually quantify data, making it easier for the reader to assess improvements over existing techniques.

4. Figures are Hard to Interpret:

Some figures, like gel electrophoresis results, can be difficult for non-experts to interpret without more explanation in the captions or labeling of DNA bands, controls, or markers.

Recommendation: Provide clearer legends, annotations, and labels on each gel lane and graph so the images become self-explanatory even for those not familiar with the experimental setup.

5. Lack of Visualized Statistical Data:

Even though the text describes some experiments as yielding "sufficient DNA" or "better results," the visual data to support these claims in the form of histograms or other statistical visualizations are minimal or unclear.

Recommendation: Include more statistical graphs (with error bars, significance markers) to strengthen the conclusions about the efficiency and reliability of the novel method.

6. Comparative Analysis Missing in Visuals:

The comparison between silica columns and the novel method is described in the text, but better comparative visuals (e.g., side-by-side photos or simplified diagrams) would help reinforce this comparison.

Recommendation: Create summary visuals that show direct comparisons between the two methods in one image (e.g., purification time, yield, cost), to make the argument more compelling.
